# Scotch Physicians and Surgeons

**Published:** 1888-06-16

**Authors:** C. G. Furley


### Scotch Physicians and Surgeons.

By C. G. Furley.

*(Concluded from p. 74.)*

### JAMES SYME.

It was in connexion with the Surgical Hospital at Minto House that the incident occurred which brought to a head the long-smouldering quarrel between Syme and Liston. The latter, meeting with a copy of the subscription-book of the hospital, wrote in it, "Don't support quackery and humbug." James Syme was not the man to endure with patience an insult such as this. He knew that he was neither quack nor humbug; he knew that Liston's words were prompted by nothing more than jealousy of a fame that promised before long to rival his own; and he determined—not, perhaps, without a certain satisfaction (for though Syme, unlike his great colleague, never forgot the bearing of a gentleman, the jealousy was mutual)—to bring him to account. He raised an action against Liston, and, though with some trouble, the latter was compelled to give a written recantation and apology, besides paying the expenses incurred by Syme in forcing him to do so—a tax which must be the bitterest drop in the cup of the defeated litigant. This incident did not tend much to improve the relations between the two surgeons, nor were these greatly bettered when in 1833 Syme was appointed Professor of Clinical Surgery at the University of Edinburgh. By virtue of this appointment he received wards in the Infirmary, and so had no need to maintain such an expensive luxury as a private hospital.

At the Infirmary Syme and Liston went on the principle of ignoring each other elaborately. There were rather too many surgeons of the first rank in Edinburgh just then. Liston was the eldest of all, and in some respects the most famous. The dash and brilliancy of his operations—surgical *tours de force*, which dazzled his students—gave him a certain pre-eminence, in which, however, John Lizars, who had, mainly by Liston's iufluence, been appointed Professor of Surgery in the Royal College of Surgeons, ran him close. Besides Liston and Lizars, there was Mr.—afterwards Sir William—Fergusson, striving to rival Syme, who was already faintly suspected of being, in spite of the lack of fuss and display in his operations, a surgeon of more originality and daring than any of the others. But while these four lived and moved and had their being in Edinburgh, there was obviously too much wealth of surgical knowledge for happiness. Therefore, when in 1835 Liston accepted the chair of Clinical Surgery in the University of London, it was an appreciable relief to the medical circles of Edinburgh. Syme, who was superior in age to Fergusson and in manners to Lizars, at once became the recognised head of the art of surgery in Scotland. By the time he had reached the age of thirty-four his position, gained not without struggle, was fully assured. Only one further important vexation was to trouble his professional career.

Liston was happier in London. He had elbow-room there, and had not to dread the encroachments of a clever surgeon but little his junior. Three hundred miles away from Syme, and no longer fearing him as a rival, his feelings towards his old colleague changed, and he began to long for a return to the friendship that had previously existed between them. It took some time for this change to be wrought, and it was not till the end of 1839 that Liston wrote a very simple and manly letter, asking for a reconciliation, and adding by way of postcript the plea—"I am not so bad as you believe me to be." Syme's reply to this is not extant; but we know that he accepted the hand thus outstretched, and that Liston visited him in Edinburgh in 1847.

It must have been gratifying to Syme to recall afterwards this renewal of the friendly companionship of youthful days, for this meeting with Liston in his own home, was the last they ever had. A few months after, all rivalry came to an end: Liston died suddenly, and Syme—destined even yet to be closely connected with him—was offered the post he had held in London. He hesitated a little, but to be Liston's successor was an honour, and the prospect of consulting practice in London was tempting. So he accepted the chair, and early in 1848 delivered his first lecture in University College.

Mr. Patrick Geddes, biologist, economist, art critic, and philanthropist, boasts himself to be "the Scotchman who went to London—and came back." He has a predecessor in this feat: James Syme, too, came back to Edinburgh within six months of quitting it. He found London disappointing. It was not that the streets were not paved with gold, but that he was not allowed to go into them and gather it. His prospects of practice were very good, but he found that the authorities of University College expected him to conduct a systematic as well as a clinical course of study, which would, combined with the position, inseparable from the clinical chair, of surgeon to the North London Hospital, render private piactice impossible. While he was meditating over the *désagremens* of this situation he was present at the summer distribution of prizes to the students of the college, when two professors, one of them an old and intimate friend of his own, were treated with insult. One of the insulted professors turned to Lord Brougham, and appealed to him to preserve order. Brougham laughed, and compared the scene to those he had often witnessed in the House of Commons. This was, for Syme, the last straw. A college where the students could openly make a mock of their teachers without rebuke was not a place for him; and that evening, after walking up and down his study for a long time, he suddenly said to Sir Robert Christison, who was then his guest, "I am going back to Edinburgh!"

The resolution thus suddenly made was soon carried out, and on July 3rd, 1848, he went home again to Edinburgh, and even to his old post in the University and Infirmary; for these had been filled only in a provisional manner, and all who had a voice in choosing occupants for them were glad to have the task of an invidious selection removed by the chance of restoring them to the man who, by popular consent, was the most competent to fill them. Syme's last words on the subject of his metropolitan experiment are given in a letter to his friend, Dr. Sharpey: "The whole expense of my London expedition is under £2,000, and promises soon to be made up. Indeed, I would gladly have paid a larger sum for the peace and comfort of being free from animosity and contention."

So he returned to spend the rest of his life in the city of his birth, his early struggles, and his ultimate success. No temptation to sacrifice happiness to ambition had power to move him again: like the anonymous brother Scot of less honourable renown, he had "tried baith," and knew which was best. Not that it can be said that he returned to peace and idleness; both of these things were equally impossible to him. He worked hard, and he had quarrels with a sadly large number of his contemporaries.

The most to be deplored of all these differences—the only other that merits reference in a sketch of this nature—is the long discord that existed between Syme and Simpson. The former had opposed Simpson's election to the chair of Midwifery, and the dislike thus engendered was maintained by those foolish people who will insist on comparing persons and things whose characteristics do not afford just data for such comparison. "Why will people go on asking which is the greater poet, Schiller or myself?" exclaimed Goethe. "They ought to be thankful to have us both." The advice was good, but it did not suppress the questioners. And in Edinburgh everybody wanted to know if Syme or Simpson was the more distinguished man, and when they came to any conclusion became violent partisans of one or the other. It is thus that enmities are made, and even when the principals—those whose names are blazoned on the rival flags—have little to do in raising the quarrel, they are compelled at last to take part in it.

One reasonable cause of coolness there certainly was. Professor Syme, whose employment of it would have been the best testimony of its value, did not take kindly to chloroform. He said, indeed, that when he went for the first time to the operating theatre of the Infirmary to see an operation in which it was used, he thought he had mistaken the place, and intruded on a meeting of presbytery, for the room was filled with ministers of the Free Church (the religious body to which Professor Simpson belonged). On seeing, however, the good results of the use of anæsthetics he accepted them gladly; the coolness between the two professors disappeared for a time, and a friendship was sealed when Sir James Simpson suffered from a severe abscess, and Mr. Syme was called in to relieve it. But, alas! a new cause of discord was yet to arise. In the year 1863 Simpson introduced the system of acupressure—that is, a method of closing blood-vessels without tying them, by means of needles pressed through their soft parts. Syme opposed the new method, and clung to the old fashion of ligatures, justifying his preference not only by argument, but by a strain of invective which seemed to express the opinion that an obstetric professor had no right to invent new methods in surgery. Sir James Simpson retorted that Professor Syme condemned acupressure without either trying it or seeing it tried, and that, therefore, he was using his position in the surgical world to depreciate unjustly a method on which he was not qualified to speak. At a later period Simpson was ready to be reconciled to his great colleague, and when both were in Dublin to receive high honours from the University there, Sir Dominic Corrigan strove to bring them together, but Syme refused to accept the proffered friendship, and the estrangement continued to the end of their lives.

Against the conservatism that marked Mr. Syme's attitude towards many developments of surgical science must be set the readiness with which he adopted the antiseptic method long before Sir Joseph Lister's ideas had won acceptance with the general public. In the beginning of 1868 he published an account of the new system in the *British Medical Journal*, which, coming from a man of his distinction, was bound to carry weight, and by the generous praise it gave to Mr. Lister showed that it was no question of personal jealousy that had ever made him withhold his approval from the novelties he had opposed.

This may be looked upon as his last contribution to the science to which his life had been devoted. On the 6th of April, 1869, he was attacked by paralysis, and though he recovered from the first shock he felt it expedient to resign his professorship and his post as surgeon to the Infirmary. For a year, however, he lived in comparative health, able to go about and to entertain his friends; even, with assistance, to perform some small operations. But in the beginning of April, 1870, another attack of paralysis was experienced, and a more serious one followed in May. For five weeks he lay helpless, and at length died calmly on the 26th of June, 1870.

From this brief sketch of his life it will be seen that James Syme was not what his countrymen call "everybody's body." He was not a universal favourite; he had warm friends and very hot foes. The life-long friendship between him and Sir Robert Christison indicates a similarity of feeling on most points; but Christison was a man of wider sympathies, of less rigid conservatism; above all, possessed of a greater sense of humour. (It would be interesting to guess how much of what passes for superior benevolence of spirit in many men is due simply to the power of seeing the comic side of a situation.) But though by the world at large his talents were respected more than his personality was loved, to those whom he knew and trusted—above all, to his children—he was unfailingly tender. Though a hard struggle with untoward circumstances and frequent injustice had forced him to adopt a plate armour of asperity, his heart beat soundly at the core, and he could with justice have claimed the benefit of Browning's protest:

"God be thanked, the meanest of His creatures
Has two soul-faces: one to fight the world with,
And one to show a woman how he loves her."

